# Metal–Organic Frameworks for Precision Phototherapy of Breast Cancer

**DOI:** 10.3390/molecules31030544

**Published:** 2026-02-04

**Authors:** Fan Qi, Haitao Ren, Beibei Bie, Qiaofeng Wang, Guodong Fan, Zhaona Liu, Huanle Fang, Chuanyi Wang

**Affiliations:** 1 School of Medicine, Xi’an Peihua University, Xi’an 710125, China; 2 Technological Institute of Materials & Energy Science (TIMES), Xijing University, Xi’an 710123, China; 3 School of Environmental Science and Engineering, Shaanxi University of Science and Technology, Xi’an 710021, China

**Keywords:** metal-organic frameworks, breast cancer, photodynamic therapy, photothermal therapy, synergistic phototherapy

## Abstract

Breast cancer remains the most common and leading cause of cancer deaths among women worldwide. The efficacy of conventional therapies is often hampered by off-target effects and multidrug resistance. Phototherapy, encompassing photodynamic therapy (PDT) and photothermal therapy (PTT), has gained significant attention due to its non-invasiveness, high spatiotemporal selectivity, and minimal side effects. However, its application is hindered by several obstacles, including the tumor hypoxic microenvironment, insufficient light penetration depth, and acquired heat resistance. Metal–organic frameworks (MOFs) have adjustable structures, enormous specific surfaces, and facile functionalization, providing an ideal platform to overcome these limitations. This review summarizes the latest research progress in the application of MOFs for precision phototherapy in breast cancer treatment. It emphasizes their role as a direct photosensitizer (PS), photothermal agent (PTA), or multifunctional nanocarrier for PDT, PTT, and synergistic phototherapy (including PDT/PTT, chemo/phototherapy, and immunotherapy/phototherapy). The design strategy and therapeutic effect of MOFs for phototherapy of breast cancer are critically discussed. In addition, the current bottlenecks and future perspectives are outlined to facilitate the clinical translation of MOF-based breast cancer treatment platforms.

## 1. Introduction

Breast cancer is the most common malignant tumor and the leading cause of cancer-related mortality among women worldwide, posing a critical challenge to global public health [1,2]. According to the latest Global Cancer Statistics, there were over 2.3 million new cases and approximately 626,679 deaths from breast cancer in 2018 [3]. Tumor classification is a key aspect of customized treatment strategies in oncology. Based on patterns of gene expression, breast cancer is divided into four subtypes: triple-negative cancer (TNBC), luminal A, luminal B, and human epidermal growth factor receptor 2 (HER2)-positive [4,5]. Each subtype exhibits different biological behaviors, prognoses, and treatment responses, which helps in selecting the most appropriate treatment strategy. Currently, the most popular clinical therapies for breast cancer include radiation, chemotherapy, and surgery [6,7,8]. However, the efficacy of these treatments is often limited by off-target effects, multidrug resistance, and the inability to completely eradicate tumors. Therefore, there is an urgent need to develop effective breast cancer treatment strategies.

**Figure 1:**
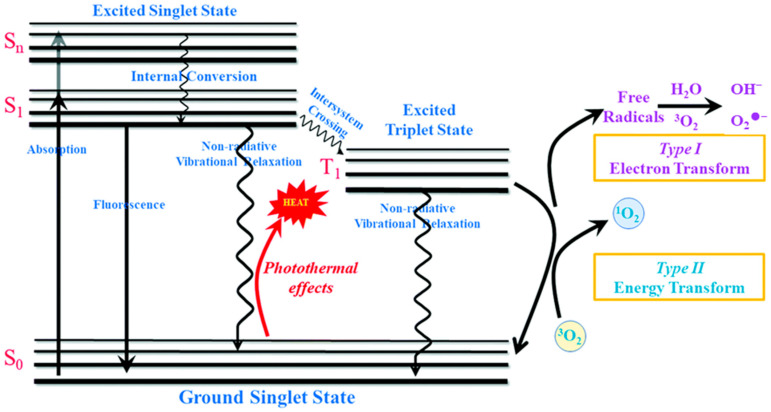
Schematic illustration of the Jablonski diagram for phototherapy [14]. Reproduced with copyright permission from Ref. [14].

Phototherapy has attracted considerable attention as an emerging treatment modality due to its favorable therapeutic effect, negligible side effects, non-invasiveness, and high spatiotemporal precision [9,10,11]. According to the treatment mechanisms, photodynamic therapy (PDT) and photothermal therapy (PTT) are two main types of phototherapy (Figure 1) [12,13,14]. Wherein, PDT mainly exploits a photosensitizer (PS) to transform molecular oxygen into cytotoxic reactive oxygen species (ROS) by electron (Type I PDT) or energy transform (Type II PDT), which in turn damages cancer cells through oxidative stress [15,16]. In addition, PTT utilizes a photothermal agent (PTA) to transform absorbed photon energy into local heat through non-radiative relaxation, which increases the temperature of the surrounding tumor tissues to directly ablate cancer cells via hyperthermia [17,18]. Thus, the efficacy of both PDT and PTT critically depends on the properties of the photoactivatable agent (PS or PTA). An ideal agent requires strong near-infrared (NIR) absorption, favorable biocompatibility, and high photostability [19,20,21]. To date, diverse PSs and PTAs based on inorganic nanoparticles, organic dyes, and carbon materials have been developed [22,23,24,25,26]. However, these PSs and PTAs have drawbacks, such as limited penetration depth, susceptibility to photobleaching and undesirable dark toxicity. In addition, the precision of phototherapy extends beyond spatiotemporal, theranostic, and combinatorial dimensions to include targeting precision, which can be achieved through further modification of a photoactivatable agent (PS or PTA) [27,28].

**Figure 2:**
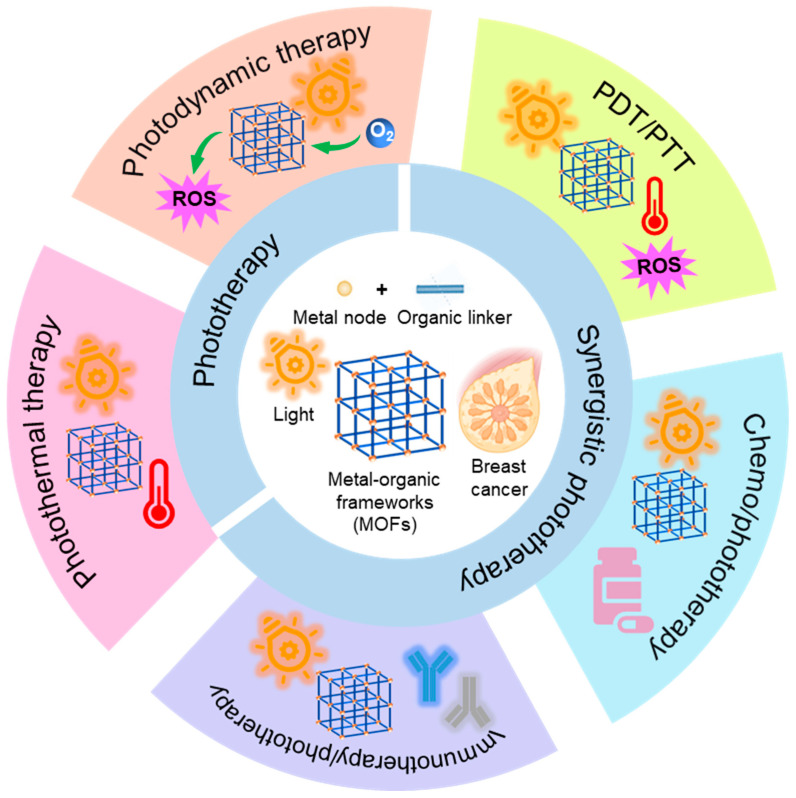
Schematic diagram of MOFs for the phototherapy of breast cancer including PDT, PTT, and synergistic phototherapy.

A class of crystalline porous materials known as metal–organic frameworks (MOFs) is formed when organic ligands (functioning as “linkers”) and metal ions or clusters (serving as “nodes”) self-assemble [29,30,31,32]. This unique hybrid composition endows MOFs with a series of exceptional properties, including ultrahigh porosity, a large specific surface, adjustable architectures, and functional diversity, which is unparalleled by many conventional nanomaterials [33,34,35,36,37]. Furthermore, MOFs, as prominent materials, are extensively utilized in diverse fields such as gas separation, catalysis, and biomedical applications [38,39]. MOFs have shown significant promise in biological applications, including bioimaging, tumor treatment, and diagnostics [16,40,41,42,43]. In particular, there has been an increasing amount of research on MOFs for the phototherapy of tumors in recent years [44,45,46,47,48]. The primary functions of MOFs in breast cancer phototherapy are highlighted in Figure 2. MOFs can function directly as PTAs or PSs by employing light-responsive building units [49]. On the other hand, they can achieve photo-responsiveness by introducing PTAs or PSs; in addition, the size-adjustable porous structures in MOFs improve photoconversion efficiency via preventing the self-aggregation of loaded PTAs or PSs [50,51]. The appropriate structural design and modifications of MOFs can modulate the light absorption and electron-transition pathway to improve phototherapy efficacy [52,53]. MOFs also serve as multifunctional platforms for combination with chemotherapy and immunotherapy [54,55]. These encouraging results highlight MOFs as promising platforms for the phototherapy of breast cancer.

In this review, we survey the latest achievements of MOFs in phototherapy, including PDT, PTT, and synergistic phototherapy, the combination of chemotherapy or immunotherapy for breast cancer. The design strategies and methods of the phototherapeutic MOFs are emphatically discussed. Furthermore, the therapeutic effects of MOFs on breast cancer utilizing phototherapy and synergistic phototherapy are outlined. Lastly, we highlight the current bottlenecks and challenges in this developing scientific field. By focusing on the extensive applications of MOFs in breast cancer phototherapy, we aim to accelerate the exploration of MOFs in breast cancer treatment, thereby improving phototherapeutic outcomes.

## 2. MOFs for PDT of Breast Cancer

The latest progress in PDT focuses on nanoparticle-based PSs, among which MOFs utilizing organic ligands like porphyrin and BODIPY are prominent, owing to their unique phototherapy potential [56,57]. It is worth noting that the coordination of these organic ligands with the metal nodes within MOFs significantly increases the ROS yield [58]. Despite being promising candidates for PDT, the efficacy of MOFs is often hampered by the hypoxic microenvironment inherent to solid tumors. Hence, researchers have designed more sophisticated MOF-based PSs and complex composite systems, which will be discussed in this section.

**Figure 3:**
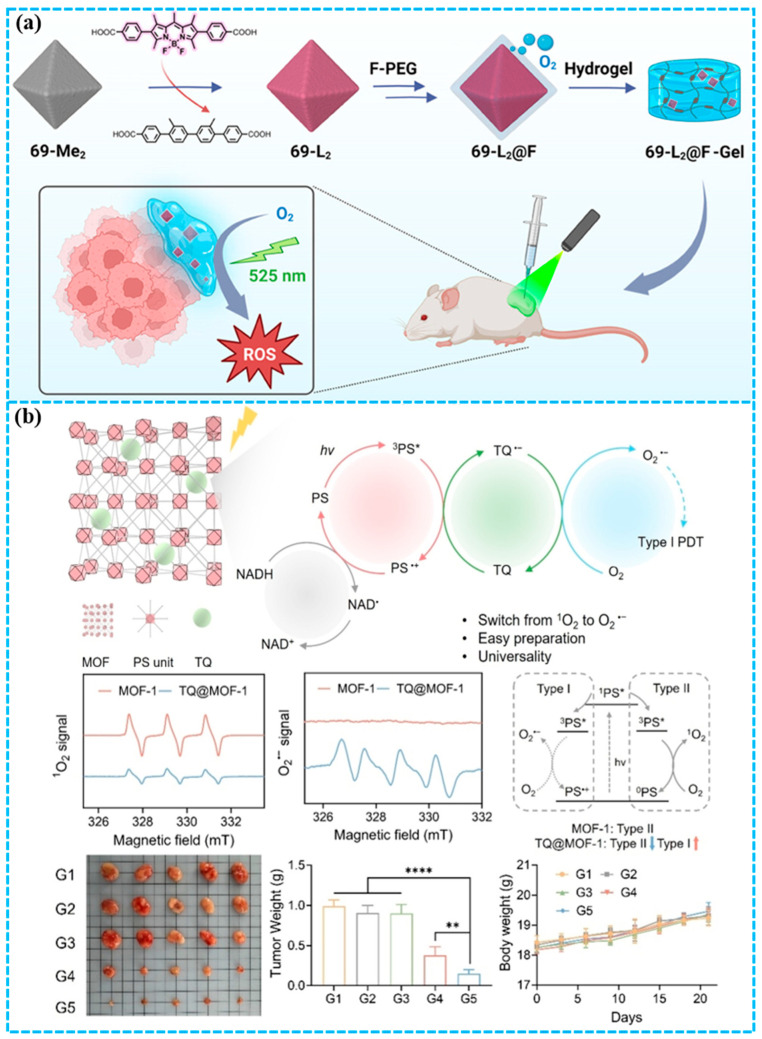
(**a**) Construction of 69-L_2_@F for the PDT of triple-negative breast cancer [59]; (**b**) design of TQ@MOF-1 NPs for the PDT of 4T1 tumors [64], Statistical significance was analyzed via one-way ANOVA test with a Tukey post hoc test, **** *p* < 0.0001, ** *p* < 0.01. Reproduced with copyright permission from Refs. [59,64].

Chen and co-workers [59] successfully constructed the first zirconium-based MOF (69-L_2_) composed of BODIPY-derived ligands through a single-crystal to single-crystal post-synthetic exchange strategy, solving the problem of BODIPY instability under harsh MOFs synthesis conditions. To further improve the PDT performance in hypoxia, fluorinated phosphate-customized methoxy poly(ethylene glycol) was modified on the outer surface of 69-L_2_, resulting in 69-L_2_@F (Figure 3a). 69-L_2_@F is an oxygen-carrying nanoplatform that simultaneously acts as a PS, thereby integrating tumor oxygenation and light-triggered ROS generation within a single agent under LED irradiation. 69-L_2_@F demonstrated a significantly enhanced PDT efficacy against MDA-MB-231 TNBC cells, which was retained even under hypoxia. Building on the positive in vitro data, 69-L_2_@F incorporated with a hydrogel was administered locally in a murine model of TNBC, eliciting remarkable antitumor efficacy in only 2 days. This work paves the way for the rational design of promising MOF-based PSs for hypoxic tumors.

In contrast to Type II PDT, Type I PDT can alleviate the stringent dependence of PDT on oxygen, generating cytotoxic ROS, such as superoxide anion (O_2_^•−^) and hydroxyl radicals (^•^OH), via an electron-transfer pathway [15,60,61,62,63]. Consequently, the development of Type I PDT holds significant promise for enhancing the therapeutic outcomes of PDT in hypoxic tumors, representing a prioritized research direction. Zhuang and co-workers [64] developed an electron-transfer strategy to convert conventional Type II MOFs into hypoxia-tolerant Type I systems by encapsulating thymoquinone (TQ) (Figure 3b). By serving as an efficient electron-transfer mediator, TQ facilitated electrons from the photoexcited MOFs ligand to oxygen, thereby establishing a dominant Type I pathway while attenuating the innate Type II process. Then, four typical porphyrin-based MOFs were prepared and loaded with TQ to successfully demonstrate the feasibility and universality of the proposed strategy. Further in vivo results indicated that TQ@MOF-1 NPs exhibited better PDT efficacy in the 4T1 tumor model compared to MOF-1 NPs. This research presents a robust and universal strategy for modulating ROS generation in PS-based MOFs, conferring hypoxic tolerance and enhanced PDT efficacy against breast cancer.

The MOF-based PDT platforms discussed primarily employ two design strategies to overcome tumor hypoxia: (1) integrating oxygen-carrying components or catalytic nanoenzymes, and (2) shifting ROS generation mechanisms from oxygen-dependent Type II pathways to oxygen-independent or less-oxygen-dependent Type I pathways via electron-transfer mediators. A common advantage of these platforms is enhanced ROS yield achieved through the porous, ordered structure of MOFs, thereby reducing PS aggregation. However, key limitations remain, including the potential complexity of composite preparation and the need for light sources capable of deeper tissue penetration. The clinical translational significance of these systems lies in demonstrating robust efficacy in more clinically representative hypoxic solid tumor models, rather than subcutaneous xenograft models.

## 3. MOFs for PTT of Breast Cancer

PTT holds distinct advantages in tumor treatment compared with PDT [18,65,66]. Unlike PDT, which can cause prolonged photosensitivity requiring strict sunlight avoidance, PTT is not activated by ambient light, significantly improving patient safety and convenience. Furthermore, as PTT’s mechanism is oxygen-independent, it presents superior therapeutic potential for hypoxic tumors.

**Figure 4:**
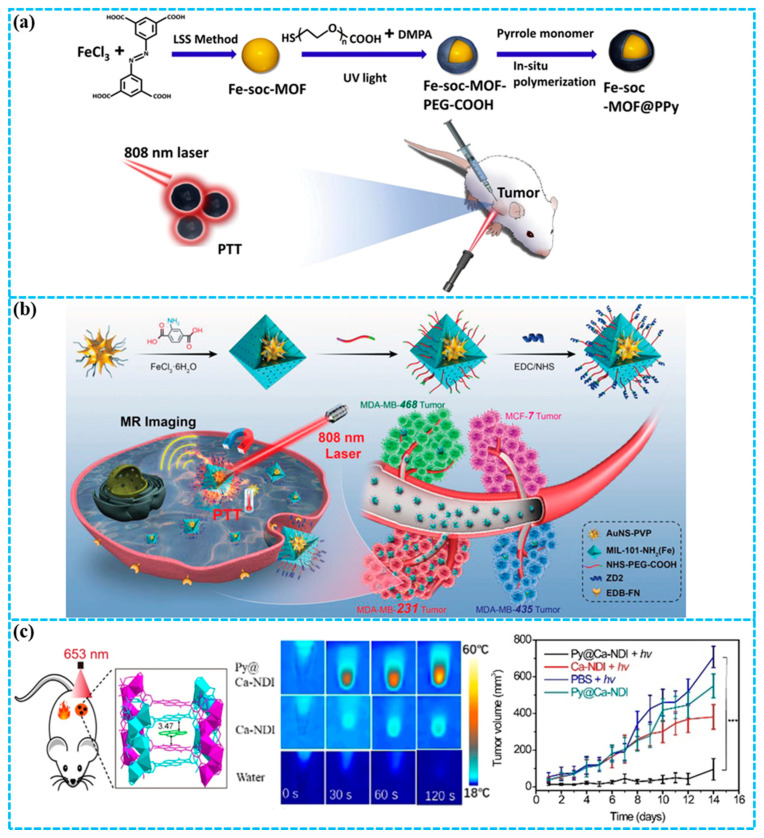
(**a**) Preparation of Fe-soc-MOF@PPy for PTT [71]; (**b**) synthesis of AuNS@MOF-ZD2 for T1-weighted MR imaging and PTT specifically targeting an MDA-MB-231 tumor (TNBC) [75]; (**c**) preparation of Py@Ca-NDI for PTT of breast cancer [81], values are means ± s.e.m. (*n* = 5 mice per group). *** *p* < 0.001. Reproduced with copyright permission from Refs. [71,75,81].

Biodegradable and low-toxicity porous iron(III) carboxylate MOFs have garnered significant attention for biomedical applications [67,68]. In particular, integrating these MOFs with organic polymers has emerged as a promising strategy [69,70]. Cai and co-workers [71] first prepared nano Fe-soc-MOF using the liquid–solid–solution method, and further constructed a multifunctional theranostic platform (Fe-soc-MOF@PPy) by integration with polypyrrole (PPy) (Figure 4a). The Fe-soc-MOF@PPy nanocomposite exhibited a photothermal conversion efficiency (PCE) of 13.9% in aqueous solution. Under 808 nm near-infrared laser irradiation, Fe-soc-MOF@PPy effectively produced heat for PTT in mice bearing 4T1 cell line-derived xenograft tumors. This study highlights the enormous potential of MOFs and functional polymers in constructing core–shell structures for synergistic diagnosis and treatment of breast cancer.

TNBC is a particularly aggressive subtype, characterized by its notably high invasiveness and metastatic potential [72,73]. By exploring versatile nanomaterials coupled with targeting ligands, an active-targeted nanoplatform could enhance the potency of TNBC theranostics. MIL-101-NH_2_(Fe) stands out among various MOFs as an excellent shell material owing to its favorable biocompatibility and inherent biodegradability. The amino groups on MIL-101-NH_2_(Fe) are extremely useful for further customization to implement active-targeted capability [74]. Zhang and co-workers [75] constructed a well-defined core–shell AuNS@MOF-ZD2 nanocomposite through a four-cycle coating process, which encapsulated a single gold nanostar (AuNS) within a MIL-101-NH_2_(Fe) shell engineered with the TNBC-targeting ZD2 peptide (Figure 4b). The prepared AuNS@MOF-ZD2 nanocomposites exhibited a remarkable PCE (40.5%). Both in vitro and in vivo studies demonstrate AuNS@MOF-ZD2’s favorable biosafety, effective T_1_-weighted magnetic resonance (MR) imaging, and PTT under low-power 808 nm laser irradiation, achieving excellent theranostic efficacy against TNBC. Notably, the precise targeting of AuNS@MOF-ZD2 toward TNBC cells (MDA-MB-231), as opposed to other breast cancer subtypes (MDA-MB-435), is due to the specific recognition mediated by the ZD2 peptide. This work provides a novel strategy for accurate theranostic treatmeant of breast cancer based on molecular classification.

Despite considerable interest in organic self-assembled charge-transfer co-crystals due to their facile synthesis and intriguing properties, their applications in biology are hampered by poor aqueous stability, inherent hydrophobicity, and water sensitivity [76,77,78,79,80]. Zeng and co-workers [81] developed an electron-deficient MOF using naphthalene diimide (NDI) as a ligand and biocompatible Ca^2+^ as metal nodes, followed by encapsulation of the electron-donor (pyrene) to produce a self-assembled co-crystal (Py@Ca-NDI) of the host–guest MOF (Figure 4c). Single-crystal analysis demonstrated that such an MOF structure facilitated a uniquely ordered D-A arrangement with a minimal intermolecular distance (3.47 Å), leading to a record-high PCE of 41.8% for Py@Ca-NDI among organic co-crystal materials. Furthermore, tumors in 4T1 mice were effectively ablated utilizing the heat generated by Py@Ca-NDI under NIR laser irradiation. This work presents a straightforward, modular, and effective method for stabilizing organic co-crystals, with potential for extension to a broad spectrum of biological applications.

Strategies for constructing PTAs based on MOFs typically involve: (1) incorporating or attaching strong NIR absorbers onto MOFs, and (2) conjugating targeting ligands to enhance tumor specificity. The modular nature of MOFs enables precise tuning of PCE through controlled donor–acceptor interactions and morphological engineering, as evidenced by record-breaking PCE in organic co-crystal@MOF systems. A recurring challenge is balancing high PCE with optimal biodegradability and clearance properties. For clinical translation, future work must prioritize developing MOF-PTA platforms that combine high efficiency in the NIR-II window with well-established long-term biosafety data.

## 4. MOFs for the Synergistic Phototherapy of Breast Cancer

Although both PDT and PTT strategies based on MOF nanoplatforms have shown promising efficacy in breast cancer treatment, there are still some limitations of each therapy. The inherent hypoxia within the tumor microenvironment severely compromises the efficacy of PDT by limiting the O_2_ supply essential for generating sufficient ROS [82]. Meanwhile, the therapeutic outcome of PTT is diminished due to acquired thermotolerance, primarily driven by the high expression of heat shock proteins in tumors [83]. Another challenge facing PDT and PTT is their inadequate light penetration depth [84]. To overcome these limitations, the integration of multiple therapy modalities within a single MOF nanoplatform for synergistic phototherapy has become an attractive strategy. Recently, MOF nanoplatforms combining phototherapy with other therapeutic methods have been developed to enhance antitumor effects [85,86]. In this section, three synergistic phototherapy strategies are prioritized: PDT/PTT, chemo/phototherapy, and immunotherapy/phototherapy. This focus is driven by their strong clinical rationale, clear mechanistic synergy, and compatibility with MOF-based nanoplatforms [44]. The latter two integrate phototherapy with cornerstone systemic treatments, whereas PDT/PTT overcomes the limitations of phototherapy itself. Critically, MOFs serve as an ideal platform for realizing these synergies due to their proven capability for precise engineering and multifunctional integration.

### 4.1. MOFs for the Synergistic PDT/PTT of Breast Cancer

**Figure 5:**
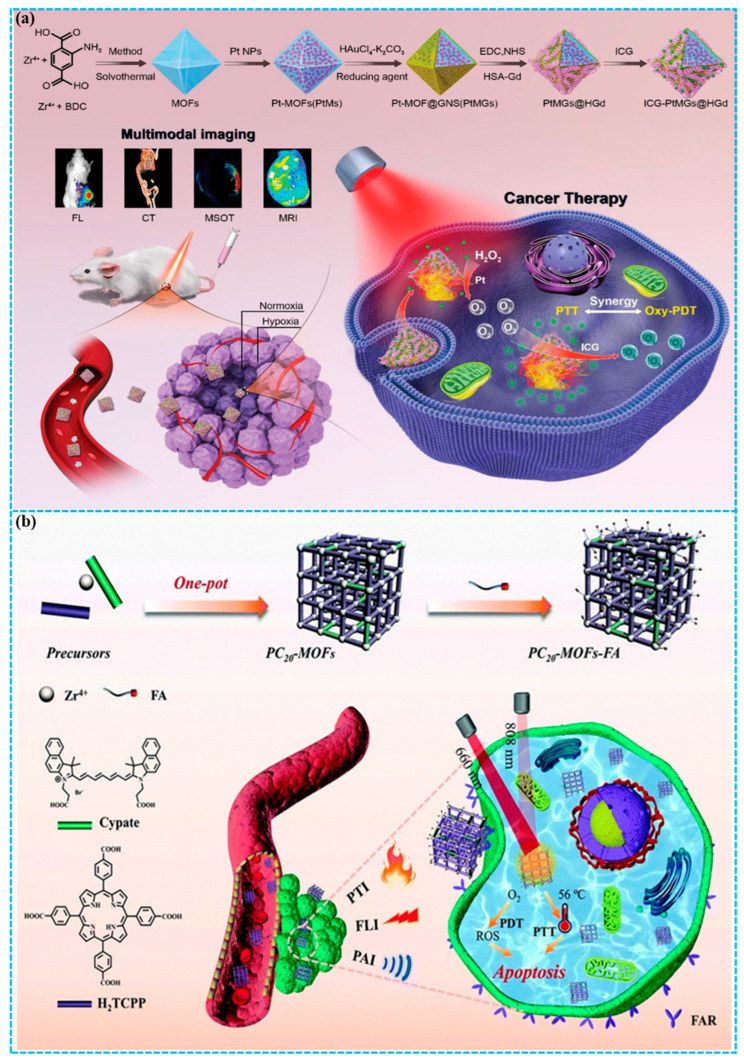
(**a**) Preparation of ICG-PtMGs@HGd nanoplatform for enhanced synergistic PDT/PTT in breast cancer [89]; (**b**) Fabrication of the PC20-MOF-FA nanoplatform for PTI/FLI/PAI trimodal imaging-guided synergistic PDT/PTT of breast cancer [90]. Reproduced with copyright permission from Refs. [89,90].

Combining PDT with PTT is an attractive method to address the limitations of each, where PTT-induced hyperthermia can improve blood flow and oxygen supply, thereby enhancing PDT efficacy, and the ROS produced by PDT can in turn destroy heat shock proteins [87,88]. Recently, MOFs have enabled multifunctional platforms for combined PDT and PTT, significantly improving therapeutic efficacy and minimizing side effects. You and co-workers [89] constructed a hypoxia-resistant and consistent O_2_ self-supplement nanoplatform (ICG-PtMGs@HGd) via a one-step method (Figure 5a). In this nanoplatform, platinum nanozyme-decorated MOFs served as the inner template, which was then coated with gold nanoshells and further functionalized with ICG and human serum albumin-gadolinium complexes, thereby achieving synergistic PDT/PTT effects and multimodal imaging. Finally, both in vitro and in vivo experiments demonstrated that the nanoplatform exhibited good biocompatibility and a potent therapeutic effect against breast cancer. The developed nanoparticle is a promising multifunctional theranostic platform for multimodal imaging-guided synergistic phototherapy of solid tumors.

Wang and co-workers [90] presented nanoscale defect-engineered porphyrin/cypate-based MOFs (PC_20_-MOFs) through a one-pot protocol for multimodal breast cancer phototheranostics (Figure 5b). The integration of porphyrin and cypate as dual ligands within the MOFs enabled trimodal imaging (FLI/PAI/PTI) to direct synergistic PDT/PTT. Furthermore, the PC_20_-MOFs were surface-modified with folic acid via coordination bonding, which endowed the resulting PC-MOFs-FA nanoplatform with active targeting capability toward tumor cells. Notably, the treatment of 4T1 tumor-bearing mice with PC20-MOFs-FA under dual-wavelength (808/660 nm) laser irradiation resulted in almost total tumor suppression (~97.2%), demonstrating the potent efficacy of synergistic PDT/PTT. For PC20-MOFs-FA, we highlight its theranostic precision, achieved through the guidance of trimodal imaging (FLI/PAI/PTI) as well as the targeting precision conferred by folic acid conjugation. This research proposes an efficient approach for integrating numerous theranostic agents into a single multivariate MOF to achieve synergy, paving the way for developing versatile nanoplatforms in cancer theranostics.

### 4.2. MOFs for the Synergistic Chemo/Phototherapy of Breast Cancer

Chemotherapy refers to the use of chemical drugs such as camptothecin, cisplatin, paclitaxel, and doxorubicin for tumor treatment [91]. It has become the most commonly adopted clinical approach for treating tumors. However, its therapeutic efficacy remains unsatisfactory, primarily attributed to a lack of tumor selectivity causing adverse effects, as well as the development of drug resistance leading to tumor recurrence [92,93]. To solve these issues, combining chemotherapy with phototherapy for tumor treatment is urgently needed. The ROS generated via PDT inhibits the expression of active efflux transporters, while the heat from PTT facilitates drug uptake by increasing membrane permeability. Together, these effects promote the accumulation of drugs within tumor cells, thereby enhancing therapeutic efficacy [94]. Moreover, PTT can notably sensitize tumors to chemotherapeutics that impair DNA repair mechanisms by inducing localized hyperthermia [95]. Therefore, the synergistic combination of chemotherapy and phototherapy represents a promising strategy for achieving superior anticancer outcomes.

**Figure 6:**
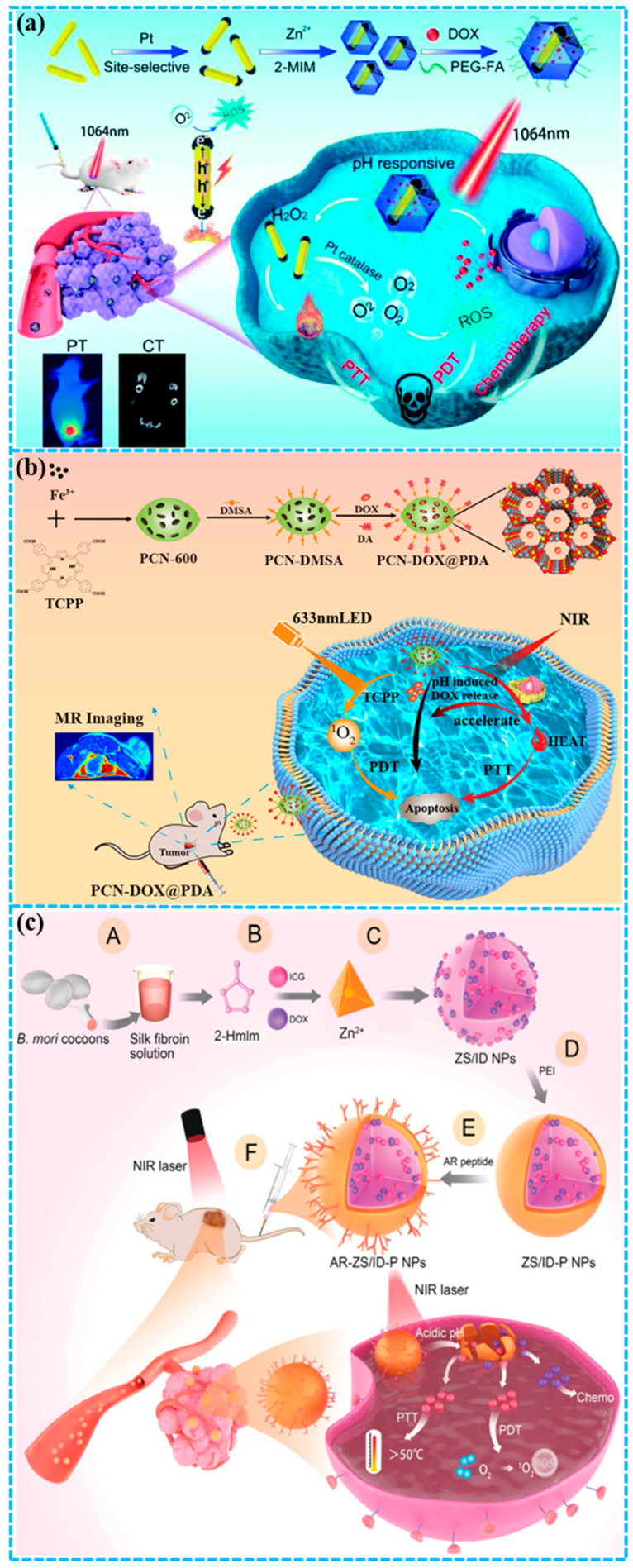
(**a**) Construction of a DOX-Pt-tipped Au@ZIF-8 nanoplatform for PT/CT imaging-guided combinatorial CT/phototherapy of breast cancer under a single NIR-II laser [96]; (**b**) preparation of PCN-DOX@PDA for MRI-guided synergistic CT/phototherapy of breast cancer [97]; (**c**) fabrication of AR-ZS/ID-P NPs for target peptide-guided synergistic CT/phototherapy of breast cancer [98]. Reproduced with copyright permission from Refs. [96,97,98].

Chen et al. [96] successfully developed a stimuli-responsive versatile nanoplatform by integrating the anticancer drug DOX and plasmonic bimetal heterostructures into a ZIF-8 matrix, resulting in the DOX-Pt-tipped Au@ZIF-8 nanocomposite for PT/CT imaging and combinatorial chemo/phototherapy (Figure 6a). Pt-tipped Au nanorod (NR) heterostructures were formed via the selective growth of Pt nanocrystals with catalase-like activity on the ends of Au NRs. The Pt-tipped Au NRs showed exceptional photothermal and photodynamic performance compared to Au NRs and Pt-coated Au NRs under 1064 nm laser irradiation, which is attributed to their more effective plasmon-induced electron–hole separation. The photothermal effect potentiates the catalytic activity of Pt, thereby raising O_2_ concentrations and alleviating hypoxia. Meanwhile, the strong NIR-II absorption and substantial-Z components (Au, Pt) in DOX-Pt-tipped Au@ZIF-8 enable its capability for PT and CT imaging. Furthermore, treatment with DOX-Pt-tipped Au@ZIF-8 and 1064 nm laser irradiation facilitates synergistic chemotherapy and PTT/PDT, resulting in complete tumor suppression in the 4T1 mouse model. The DOX-Pt-tipped Au@ZIF-8 nanocomposite is designed to alleviate hypoxia, exemplifying a precise combinatorial approach that enhances therapeutic efficacy. This study establishes a strategy for developing a multifunctional nanoplatform that enables synergistic chemo/phototherapy guided by PT/CT imaging under a single laser.

Chen and co-workers [97] developed a NIR and pH dual-responsive multimodal platform (PCN-DOX@PDA) for diagnosis and treatment (Figure 6b). PCN-600 is a porous MOF incorporating the photosensitive ligand tetrakis(4-carboxyphenyl)porphyrin (TCPP) and also serves as a carrier for PDA and DOX. NIR irradiation is triggered the generation of cytotoxic ^1^O_2_ and a potent photothermal effect in PCN-DOX@PDA, effectively killing tumor cells. This robust photothermal performance was attributed to the great enhancement of NIR absorption by the PDA. PCN-DOX@PDA enabled DOX to undergo the intelligent release in response to both a weakly acidic tumor microenvironment and thermal stimulation from NIR irradiation. Furthermore, owing to the Fe^3+^ central ion in PCN, PCN-DOX@PDA accomplished tumor theranostics via magnetic resonance imaging (MRI)-guided tumor chemotherapy and synergistic PDT/PTT. Experiments in vivo using the 4T1 mouse model demonstrated that treatment with PCN-DOX@PDA NPs followed by dual-wavelength irradiation (633 nm LED + 808 nm laser) achieved complete tumor eradication and prevented relapse, confirming the potent antitumor capability of the designed trimodal synergistic therapy.

Chen and colleagues [98] proposed a nanoscale ZIF-8-based theranostic agent engineered with pH sensitivity and tumor-targeting capability for combined chemo/phototherapy (Figure 6c). Silk fibroin was first introduced as a bio-template into the 2-methylimidazole solution. Following the subsequent addition of DOX and ICG, the one-pot formation of Zn^2+^-triggered ZIF-8 (ZS/ID NPs) was initiated. The NPs were coated with polyethylenimine (PEI) to realize long-time stability and provide abundant amine groups for coupling, producing ZS/ID-P NPs. The MCF-7 breast tumor-targeting peptide AREYGTRFSLIGGYR (AR peptide) was then conjugated to this PEI layer to generate AR-ZS/ID-P NPs for selective breast tumor treatment. Upon intravenous injection, AR-ZS/ID-P NPs specifically accumulated in breast tumors and showed superior anticancer efficacy compared to other groups. This enhanced effect resulted from the synergistic combination of ICG-mediated PDT/PTT and DOX-based chemotherapy, while avoiding side effects.

### 4.3. MOFs for the Synergistic Immunotherapy/Phototherapy of Breast Cancer

Immunotherapy, as an emerging therapeutic strategy, aims to activate the immune system to precisely recognize and kill tumor cells, while effectively inhibiting tumor metastasis and recurrence [99,100]. During immunotherapy, dying tumor cells release specific tumor-associated antigens (TAAs), which are subsequently taken up, processed, and delivered by antigen-presenting cells (APCs) via major histocompatibility complexes (MHCs) to activate external T cells. This is followed by the rapid proliferation and differentiation of the activated T cells into large numbers of cytotoxic T lymphocytes (CTLs), which kill tumor cells. Concurrently, a portion of the tumor-killing T cells differentiate into memory T cells, thereby establishing long-term immunity against the tumor [101,102]. However, immunotherapy is effective only in a subset of cancer patients due to individual variability of the immune system. In addition, its clinical application is hampered by several challenges, including organ toxicity, tumor heterogeneity, and weak immunogenicity [103]. It is noteworthy that the combination of immunotherapy and phototherapy elicits a potent synergistic effect in cancer treatment, which is mediated by two critical mechanisms [92,104,105]. Firstly, phototherapy efficiently generates TAAs in situ and converts immune “cold” tumors into “hot” tumors to initiate a strong antitumor immune response. Secondly, immunotherapy utilizes the activated immune state to systematically eradicate primary and metastatic lesions, thereby preventing recurrence and improving the overall therapeutic efficacy.

**Figure 7:**
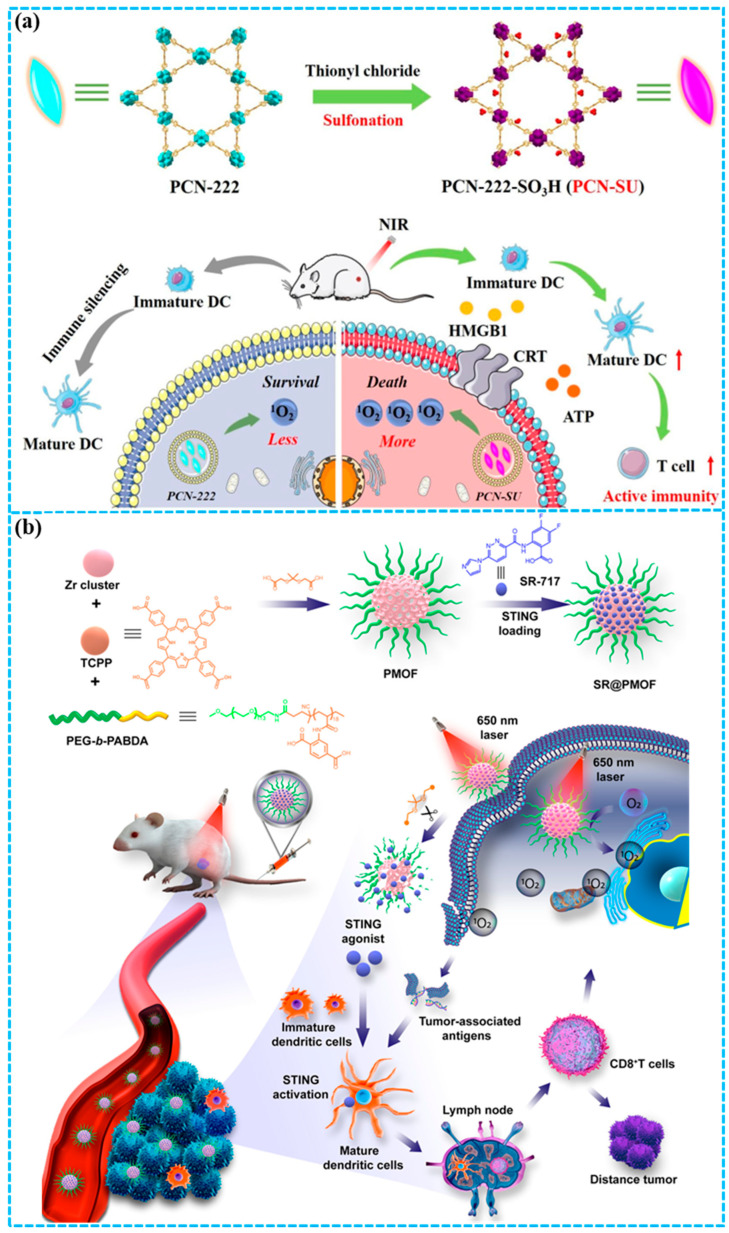
(**a**) Preparation of PCN-SU for photodynamic-immunotherapy of breast cancer [106]; (**b**) construction of SR-717-loaded PMOF NPs for photodynamic-immunotherapy of breast cancer [107]. Reproduced with copyright permission from Refs. [106,107].

Li and co-workers [106] developed a spindle-shaped PCN-222-SO_3_H (PCN-SU) that enhanced NIR absorption through sulfonation (Figure 7a). The presented sulfonate anions formed strong intramolecular hydrogen bonds with the TCPP ligands, inducing deformation of the porphyrin rings. This structural distortion resulted in a reduced energy gap between the HOMO and LUMO, thereby enhancing NIR absorption. PCN-SU achieved an increased ^1^O_2_ yield under NIR irradiation compared to PCN-222, owing to its superior photoactive performance. Furthermore, a significantly stronger immunogenic cell death (ICD) induction via ^1^O_2_ was realized by sulfonated PCN-SU under NIR irradiation compared to the PCN-222. The combination of anti-PD-1 and PCN-SU potentiated the infiltration of CTLs into tumors upon NIR irradiation, thereby showing an outstanding inhibition rate of 99.6% for 4T1 tumor-xenograft BALB/c mice in vivo. This work presented a sulfonation strategy to enhance PDT and related combination therapies by boosting the photoactivity of porphyrin-based delivery systems.

Zhou and co-workers [107] constructed polymeric MOF (PMOF) NPs that combine PDT with enhanced STING activation to boost immunotherapy efficacy (Figure 7b). The PMOF NPs with poly(ethylene glycol) (PEG) shells were synthesized by coordinating zirconyl chloride with a ligand mixture of the block copolymer PEG-*b*-PABDA, *meso*-tetra(carboxyphenyl)porphyrin (TCPP), and thioketal diacetic acid. Furthermore, the STING agonist SR-717 was introduced into the porous PMOF structure, obtaining SR@PMOF NPs with high stability under physiological environments. Upon intravenous injection and tumor accumulation, localized light irradiation triggered efficient ^1^O_2_ generation by TCPP, which induced apoptosis and thereby led to the release of fragmented DNA and TAAs. Meanwhile, the thioketal bonds were cleaved by the generated ^1^O_2_, enabling the disruption of the PMOF structure and rapid release of SR-717. The combination of SR-717 and PDT synergistically enhanced antitumor immunity via a photodynamic-immunotherapy approach, a process mediated by the reversal of the immunosuppressive tumor microenvironment and potentiation of endogenous STING activation, thereby effectively suppressing the growth of both primary and distant 4T1 tumors.

The design of MOF-based synergistic platforms consistently centers on multifunctional integration: encapsulating or co-loading chemotherapeutic agents and immunomodulators within a single MOF matrix, or combining PDT and PTT reagents. Introducing targeting groups and stimulus-responsive coatings through surface engineering represents a common strategy for achieving spatiotemporally controlled activation. Their collective advantage is enhanced therapeutic efficacy through mechanisms like hypoxia relief, heat shock protein suppression, and immune activation, often complemented by multimodal imaging capabilities. Despite encouraging preclinical results, major translational challenges include the scalability and reproducibility of these multifunctional nanocomposites, management of potential synergistic toxicity, and regulatory pathways for combination products. Future designs should prioritize simplification without sacrificing functionality to advance clinical applications.

## 5. Conclusions and Perspectives

The construction of therapeutic MOFs for breast cancer phototherapy is a fast-evolving and promising field. As displayed in Table 1, we have comprehensively outlined the latest developments of MOF-based precision phototherapy for breast cancer, focusing on their application in PDT, PTT, and synergistic phototherapy (including PDT/PTT, chemo/phototherapy and immunotherapy/phototherapy). MOFs have become ideal building blocks for efficient and multifunctional phototherapy platforms because of their variable composition, high porosity, easily modifiable surfaces, and outstanding optical properties. Through rational design, MOFs serve as a multifunctional platform capable of acting as direct PSs/PTAs or carriers, thereby promoting tumor-specific delivery, imaging-guided therapy, and synergistic therapy, especially for aggressive forms like TNBC. Although MOF-based phototherapy platforms have made significant advancements in preclinical research, their clinical translation faces key challenges and opportunities.

**Table 1:** Typical examples of MOFs for phototherapy of breast cancer.

MOF	Organic Ligand	Additional Agents	Irradiation Wavelength	Therapeutic Modality	Refs.
Zr-MOFs	BODIPY	F-PEG	525 nm	PDT	[59]
PCN-224	TCPP	TQ	60 mW/cm^2^	PDT	[64]
Fe-soc-MOF	H_4_-ABTC	PPy	808 nm	PTT	[71]
MIL-101-NH_2_(Fe)	2-APDC	ZD2/AuNS	808 nm	PTT	[75]
Ca-NDI	H_4_BINDI	Pyrene	653 nm	PTT	[81]
Pt-MOFs	NH_2_-BDC	HSA-Gd/ICG	808 nm	PDT/PTT	[89]
PC_20_-MOFs	TCPP/Cypate	FA	660/808 nm	PDT/PTT	[90]
ZIF-8	MIM	DOX-Pt-tipped Au	1064 nm	Chem/PDT/PTT	[96]
PCN-600	TCPP	DOX/PDA	633 nm	Chem/PDT/PTT	[97]
ZIF-8	MIM	DOX/ICG/AR	808 nm	Chem/PDT/PTT	[98]
PCN-222	TCPP	aPD-1	730 nm	Immunotherapy/PDT	[106]
PMOFs	TCPP	SR-717	650 nm	Immunotherapy/PDT	[107]

First, although current research primarily focuses on the therapeutic efficacy of MOFs, their long-term biosafety is a major obstacle to clinical translation [108,109]. Future work requires a comprehensive assessment of the acute and chronic toxicity of MOF components (metal ions and organic ligands) and their degradation products. It is notable that investigating the in vivo fate of stable MOFs (e.g., Zr-based) and those containing toxic metal compoents (e.g., Cd, Pd) is crucial, with a focus on their biodegradation, metabolic pathways, and accumulation in reticuloendothelial system organs [110]. Strategies to minimize the inherent toxicity associated with MOFs, while optimizing their size, morphology, and targeting efficacy, are critical for achieving maximal tumor accumulation with minimal off-target effects. Second, despite tumor accumulation improvement through passive (EPR effect) and active (e.g., folate, RGD peptides, or ZD2 peptides modification) targeting, the heterogeneous tumor microenvironment and dense extracellular matrix significantly hinder the deep penetration of MOFs. To address this problem, future research should focus on customizing the nanoscale architecture of MOFs, developing smart, multi-stimulus-responsive systems, and exploring external field-driven mechanisms. Third, the shallow penetration of visible light represents the most critical limiting factor in phototherapy. Therefore, a key direction in MOF design is developing systems activated within the near-infrared II (NIR-II) window (1000–1700 nm) [111]. The NIR-II window enables greater tissue penetration and higher maximum permissible exposure [112,113]. By rational bandgap engineering, such as introducing electron donor–acceptor pairs and utilizing *J*-aggregates, the light absorption of MOFs can be readily shifted to the NIR-II region, thereby enabling effective treatment of deeper-seated tumors.

In conclusion, MOFs offer a multifunctional and highly attractive platform for precise phototherapy in breast cancer. Through the integration of diagnostic and therapeutic functions and the realization of synergistic therapy, they overcome the key limitations of traditional phototherapy and broader cancer treatment modalities. Despite numerous challenges in clinical translation, the continuous integration of materials science, nanotechnology, and biomedical engineering is key to overcoming these obstacles. There is reason to believe that advanced phototherapy strategies based on MOFs will ultimately bring new hope to patients and hold an important position in the field of tumor treatment.

## Data Availability

No new data were created or analyzed in this study. Data sharing is not applicable to this article.

## Abbreviations

The following abbreviations are used in this manuscript:

TNBC	Triple-Negative Breast Cancer
PDT	Photodynamic Therapy
PTT	Photothermal Therapy
PS	Photosensitizer
ROS	Reactive Oxygen Species
PTA	Photothermal Agent
NIR	Near-Infrared
MOFs	Metal–Organic Frameworks
PCE	Photothermal Conversion Efficiency
MR	Magnetic Resonance
TAAs	Tumor-Associated Antigens
APCs	Antigen-Presenting Cells
MHCs	Major Histocompatibility Complexes
CTLs	Cytotoxic T Lymphocytes
ICD	Immunogenic Cell Death
NIR-II	Near-Infrared II
